# Exploiting an early warning Nomogram for predicting the risk of ICU admission in patients with COVID-19: a multi-center study in China

**DOI:** 10.1186/s13049-020-00795-w

**Published:** 2020-10-27

**Authors:** Yiwu Zhou, Yanqi He, Huan Yang, He Yu, Ting Wang, Zhu Chen, Rong Yao, Zongan Liang

**Affiliations:** 1 Department of Emergency Medicine, Emergency Medical Laboratory, West China Hospital, Sichuan University, Chengdu, 610041 Sichuan China; 2 Disaster Medical Center, Sichuan University, No.37 Guoxue Roud, Chengdu, 610041 Sichuan China; 3 Department of Respiratory and Critical Care Medicine, West China Hospital, Sichuan University, No.37 Guoxue Roud, Chengdu, 610041 Sichuan China; 4 Public Health Clinical Center of Chengdu, Chengdu, 610000 China

**Keywords:** Coronavirus disease 2019, Nomogram, ICU admission, Prediction, Early warning

## Abstract

**Background:**

Novel coronavirus disease 2019 (COVID-19) is a global public health emergency. Here, we developed and validated a practical model based on the data from a multi-center cohort in China for early identification and prediction of which patients will be admitted to the intensive care unit (ICU).

**Methods:**

Data of 1087 patients with laboratory-confirmed COVID-19 were collected from 49 sites between January 2 and February 28, 2020, in Sichuan and Wuhan. Patients were randomly categorized into the training and validation cohorts (7:3). The least absolute shrinkage and selection operator and logistic regression analyzes were used to develop the nomogram. The performance of the nomogram was evaluated for the C-index, calibration, discrimination, and clinical usefulness. Further, the nomogram was externally validated in a different cohort.

**Results:**

The individualized prediction nomogram included 6 predictors: age, respiratory rate, systolic blood pressure, smoking status, fever, and chronic kidney disease. The model demonstrated a high discriminative ability in the training cohort (C-index = 0.829), which was confirmed in the external validation cohort (C-index = 0.776). In addition, the calibration plots confirmed good concordance for predicting the risk of ICU admission. Decision curve analysis revealed that the prediction nomogram was clinically useful.

**Conclusion:**

We established an early prediction model incorporating clinical characteristics that could be quickly obtained on hospital admission, even in community health centers. This model can be conveniently used to predict the individual risk for ICU admission of patients with COVID-19 and optimize the use of limited resources.

## Background

Novel coronavirus disease 2019 (COVID-19) is a contagious disease first reported in Wuhan, Hubei, China, with a rapidly spreading outbreak [1, 2]. According to the World Health Organization (WHO), there were more than 4,993,470 confirmed cases reported worldwide (nearly all countries and regions) and more than 327,738 deaths of the infected patients as of May 23, 2020 [3]. Currently, the number of patients with COVID-19 has been rapidly increasing in the United States, Europe, Russia, and Latin America. The infection appears to demonstrate a human-to-human transmission via droplet, aerosol, fecal, or direct contact, with an incubation period of ≥1–14 days [4]. COVID-19 infection has been reported in patients of all ages, but a higher mortality rate is being noticed in older adults and those with comorbidities of hypertension, cardiovascular disease, chronic kidney disease (CKD), diabetes, and chronic respiratory disease [5]. Obesity may also be a risk factor for respiratory failure leading to invasive mechanical ventilation in patients with COVID-19 patients [6]. The disease manifestation at presentation has been generally similar, with mild flu-like symptoms being the most frequent indication. Moreover, the most common symptoms in the general population have been fever, cough, dyspnea, and myalgia or fatigue. Nevertheless, certain patients might rapidly develop acute respiratory failure, multiple organ failure, and other fatal complications. To date, no specific treatment for COVID-19 has been fully developed.

Despite public health responses aimed at containing the disease and delay its spread, the outbreak has led to an increase in the demand for medical resources, while the medical staff themselves could also get infected. To reduce the burden on the healthcare system and provide optimal care for patients, an effective prognosis assessment of the disease is needed. A predictive model that combines multiple variables or features to estimate the risk of an infected person’s poor outcomes can assist healthcare staff in classifying patients based on the severity when allocating limited medical resources [7]. Some predictive models (e.g., Pneumonia Severity Index [PSI], CURB-65, Rapid Emergency Medicine Score [REMS], etc.) are already being used in COVID-19 patients. An earlier study about COVID-19 found that the PSI performed better than CURB-65 in predicting mortality [8]. Another study showed that the REMS could provide emergency clinicians with an effective adjunct risk stratification tool for critically ill patients with COVID 19, especially for the patients aged < 65 years. When REMS parameters cannot be completed in the emergency department, Modified Early Warning Score (MEWS), which also has a high negative predictive value (NPV) for screening, is also a second option for COVID-19 patients; the prediction accuracy of MEWS is acceptable [9]. A recent study demonstrated that the rate of severe cases had a significant regional difference [10]. Therefore, in this study, we aimed to describe the clinical characteristics of patients with confirmed COVID-19 in different cities of China where the outbreak risk levels have been different and construct an early warning prediction nomogram model incorporating clinical characteristics to identify the risk of patients with poor prognosis. The prediction nomogram considers admission to ICU as the outcome rather than patients with a poor prognosis. This nomogram contains some factors that can be obtained quickly but does not include laboratory examination data, which may help provide appropriate supportive treatment in advance and reduce the probability of severe COVID-19.

## Methods

### Patients

This retrospective, multi-center study was approved by the Ethics Committee of West China Hospital. Considering the retrospective nature of the study, written informed consent was waived by the Ethics Commission of the designated hospital for emerging infectious diseases. The study included data of consecutive patients hospitalized with laboratory-confirmed COVID-19, as reported to the National Health Commission, between January 2 and February 28, 2020. The data cutoff for the study was March 14, 2020. COVID-19 diagnosis was confirmed by high-throughput sequencing or real-time reverse-transcriptase polymerase chain reaction (RT-PCR) of nasal and pharyngeal swab specimens [11]. All the study patients were diagnosed as having COVID-19 in accordance with the WHO interim guidance [12]. Based on published articles on nomograms [13, 14], the primary cohort patients were subsequently randomly assigned, using a simple random splitting method in the R version 3.5.1 and the “caret” package, in a 7:3 ratio to the training or validation set.

### Demographical and risk variables

The following data were obtained from the electronic medical records of the patients: demographics, clinical signs on admission, clinical symptoms, clinical risk factors, and exposure to infection. Demographic data were age, sex, alcohol intake status, smoking status, obesity, and the time between the onset of symptoms to admission. The onset time of clinical symptoms was defined as prior to the first visit to the hospital. Exposure to infection was defined as exposure to Wuhan (including Wuhan residency, travel history to Wuhan, or contact with people from Wuhan) or other COVID-19–affected areas (residency, travel history, or contact with people from these areas) or exposure to patients with COVID-19. The risk of exposure to infection changed as the relevant definitions in the COVID-19 guidelines of the National Health Commission of the People’s Republic of China were modified. If data were missing from the records or clarification was needed, data were obtained by direct communication with the attending physicians or other healthcare providers. A team of experienced clinicians reviewed, abstracted, and cross-checked the data. Each record was checked independently by 2 clinicians. The clinical and demographic features of the study cohort are summarized in Table 1.

**Table 1:** Baseline characteristics of patients infected with COVID-19

	Training Cohort (%)			Validation Cohort (%)		
Characteristic	ICU (*N* = 68)	Non-ICU (*N* = 695)	*P*	ICU (*N* = 29)	Non-ICU (*N* = 295)	*P*
Age, median (IQR), years	66.5(51–76)	50(36–63)	0.000	65(51–76)	49(38–63)	0.000
Gender			0.016			0.066
Male	43(63.2)	328(47.2)		19(65.5)	135(45.8)	
Female	25(36.8)	367(52.8)		10(34.5)	160(54.2)	
Temperature on Admission			0.317			0.021
≤ 36.1	3(0.6)	45(6.5)		6(20.7)	14(4.7)	
36.2–38	56(82.4)	593(85.3)		19(65.5)	251(85.1)	
≥ 38.1	9(1.2)	57(8.2)		4(13.8)	30(10.2)	
Heart Rate, bmp			0.370			0.001
< 100	52(76.5)	568(81.7)		16(55.2)	242(82)	
≥ 100	16(23.5)	127(18.3)		13(44.8)	53(18)	
Respiratory Rate			0.000			0.000
< 22	36(52.9)	605(87.1)		14(48.3)	235(79.7)	
≥ 22	32(47.1)	90(12.9)		15(51.7)	60(20.3)	
Systolic Blood Pressure, mmHg			0.322			0.554
≤ 100	4(5.9)	20(2.9)		0(0)	12(4.1)	
> 100	64(94.1)	675(97.1)		29(100)	283(95.9)	
Drinking			0.311			1.000
Former and/or Current	54(79.4)	590(84.9)		25(86.2)	251(85.1)	
Never	14(20.6)	105(15.1)		4(13.8)	44(14.9)	
Smoking			0.005			0.766
Former and/or Current	49(72.1)	596(85.8)		26(89.7)	253(85.8)	
Never	19(27.9)	99(14.2)		3(10.3)	42(14.2)	
Time interval from the onset of symptoms to admission			0.306			1.000
> 7 days	16(23.5)	123(17.7)		5(17.2)	51(17.3)	
≤ 7 days	52(76.5)	572(82.3)		24(82.8)	244(82.7)	
Obesity			1.000			1.000
Yes	0(0)	4(0.6)		0(0)	2(0.7)	
No	68(100)	691(99.4)		29(100)	293(99.3)	
Symptoms						
Fever			0.005			0.580
Yes	54(79.4)	427(61.4)		21(72.4)	193(65.4)	
No	14(20.6)	268(38.6)		8(27.6)	102(34.6)	
Cough			0.075			1.000
Yes	48(70.6)	408(58.7)		19(65.5)	192(65.1)	
No	20(29.4)	287(41.3)		10(34.5)	103(34.9)	
Dyspnea			0.000			0.004
Yes	26(38.2)	126(18.1)		12(41.4)	51(17.3)	
No	42(61.8)	569(81.9)		17(58.6)	244(82.7)	
Fatigue			0.029			0.336
Yes	34(50)	249(35.8)		14(48.3)	110(37.3)	
No	34(50)	446(64.2)		15(51.7)	185(62.7)	
Sore Throat			1.000			0.205
Yes	8(11.8)	78(11.2)		0(0)	25(8.5)	
No	60(88.2)	617(88.8)		29(100)	270(91.5)	
Nasal Discharge			0.501			0.471
Yes	1(1.5)	27(3.9)		0(0)	14(4.7)	
No	67(98.5)	668(96.1)		29(100)	281(95.3)	
Wheeze			0.285			0.000
Yes	10(14.7)	68(9.8)		10(34.5)	26(8.8)	
No	58(85.3)	627(90.2)		19(65.5)	269(91.2)	
Chest Distress			0.687			1.000
Yes	13(19.1)	114(16.4)		7(24.1)	68(23.1)	
No	55(80.9)	581(83.6)		22(75.9)	227(76.9)	
Muscle and Joint Pain			0.848			1.000
Yes	8(11.8)	71(10.2)		3(10.3)	31(10.5)	
No	60(88.2)	624(89.8)		26(89.7)	264(89.5)	
Headache			0.749			0.794
Yes	3(4.4)	43(6.2)		3(10.3)	21(7.1)	
No	65(95.6)	652(93.8)		26(89.7)	274(92.9)	
Nausea and Vomiting			1.000			1.000
Yes	2(2.9)	25(3.6)		1(3.4)	13(4.4)	
No	66(97.1)	670(96.4)		28(96.6)	282(95.6)	
Diarrhea			1.000			1.000
Yes	6(8.8)	64(9.2)		4(13.8)	36(12.2)	
No	62(91.2)	631(90.8)		25(86.2)	259(87.8)	
Comorbidities						
Asthma			0.790			1.000
Yes	0(0)	8(1.2)		0(0)	3(1)	
No	68(100)	687(98.8)		29(100)	292(99)	
Chronic Obstructive Pulmonary Disease			0.112			0.934
Yes	4(5.9)	14(2)		1(3.4)	4(1.4)	
No	64(94.1)	681(98)		28(96.6)	291(98.6)	
Hypertension			0.011			0.410
Yes	26(38.2)	163(23.5)		9(31)	66(22.4)	
No	42(61.8)	532(76.5)		20(69)	229(77.6)	
Chronic Respiratory Disease			0.003			0.165
Yes	7(10.3)	19(2.7)		2(6.9)	4(1.4)	
No	61(89.7)	676(97.3)		27(93.1)	291(98.6)	
Cardiovascular System Disease			0.000			0.566
Yes	14(20.6)	43(6.2)		3(10.3)	17(5.8)	
No	54(79.4)	652(93.8)		26(89.7)	278(94.2)	
Chronic Kidney Disease			0.000			0.000
Yes	6(8.8)	8(1.2)		4(13.8)	3(1)	
No	62(91.2)	687(98.8)		25(86.2)	292(99)	
Chronic Liver Disease			1.000			0.624
Yes	5(7.4)	50(7.2)		3(10.3)	18(6.1)	
No	63(92.6)	645(92.8)		26(89.7)	277(93.9)	
Cerebrovascular Disease			0.326			–
Yes	2(2.9)	6(0.9)		–	–	
No	66(97.1)	689(99.1)		–	–	
Autoimmune Disease			0.013			1.000
Yes	4(5.9)	8(1.2)		0(0)	5(1.7)	
No	64(94.1)	687(98.8)		29(100)	290(98.3)	
Hematological Disease			0.149			0.425
Yes	1(1.5))	0(0)		1(3.4)	1(0.3)	
No	67(98.5)	695(100)		28(96.6)	294(99.7)	
Stroke History			0.015			0.802
Yes	5(7.4)	13(1.9)		1(3.4)	3(1)	
No	63(92.6)	682(98.1)		28(96.6)	292(99)	
Malignancy			0.341			1.000
Yes	3(4.4)	13(1.9)		1(3.4)	8(2.7)	
No	65(95.6)	682(98.1)		28(96.6)	287(97.3)	
Diabetes			0.064			0.812
Yes	14(20.6)	83(11.9)		2(6.9)	30(10.2)	
No	54(79.4)	612(88.1)		27(93.1)	265(89.8)	
Exposure to source of transmission within past 14 days						
Recently visited COVID-affected area			0.266			0.921
Yes	63(92.6)	606(87.2)		26(89.7)	257(87.1)	
No	5(7.4)	89(12.8)		3(10.3)	38(12.9)	
Contact history of COVID-19			0.019			0.044
Yes	12(17.6)	224(32.2)		3(10.3)	88(29.8)	
No	56(82.4)	471(67.8)		26(89.7)	207(70.2)	

### Definition of outcomes

The severity of COVID-19 during hospitalization was determined according to the American Thoracic Society guidelines for community-acquired pneumonia [15]. The primary outcome was defined as admission to the intensive care unit (ICU), which was similar to other studies associated with severe infectious diseases [15, 16].

### Feature selection

The training cohort, which was also used for variable selection and risk model development, comprised 763 patients hospitalized with COVID-19. As described in Table 1, 37 variables were included in the selection process. The least absolute shrinkage and selection operator (LASSO) method, which is suitable for analyzing high-dimensional data, was used to select the most significant predictive features [17, 18]. Features with non-zero coefficients in the LASSO regression model were selected in the forward stepwise logistic regression model [19]. The features considered were the odds ratio (OR) with 95% confidence interval and two-tailed *p* values. Variables with a *p*-value < 0.1 in the univariate analysis and potential significance in the multivariate analysis were included in the logistic regression analysis. The forward selection procedure was used to develop a parsimonious model to predict ICU admission for COVID-19 in our cohort.

### Development and validation of an individualized prediction model

Nomogram is a statistical model useful for risk assessment. A predictive nomogram was developed using the independent factors selected by LASSO to generate a combined indicator to estimate ICU admission for COVID-19. The nomogram can be used as a quantitative tool for physicians to assess the individual probability of ICU admission. Furthermore, the created nomogram was submitted to external validation, and the total score for each nodule was calculated. The nomogram was constructed using the total score as a factor.

### Apparent performance of the nomogram in training and validation cohorts

Adequate discrimination and calibration were performed to test and validate the prognostic accuracy of the nomogram model [20]. Discrimination was quantified using Harrell’s concordance index (C-index), in which an absolute value close to 1 indicates the strong predictive ability of the model. The nomogram was further validated by bootstrapping (1000 bootstrap replicates) to calculate the corrected C-index. Calibration plots were developed to assess the predictive accuracy and agreement between the predicted and observed disease severity. Decision curve analyzes (DCAs) were performed to assess the clinical usefulness of the nomogram. The net benefit was calculated by subtracting the proportion of patients with false-positive results from that of patients with true-positive results and by weighing the relative risk of an intervention compared with the adverse effects of unnecessary intervention. The precision of the predictions was evaluated using the area under the receiver-operating-characteristic curve (AUC). Two-sided *p* values < 0.05 indicated a statistically significant difference.

### Statistical analysis

Continuous variables were expressed as median and interquartile range. Categorical variables were expressed as absolute values and percentages. The medians of continuous variables were compared using independent group *t*-tests for normally distributed data and the Mann–Whitney test for non-normally distributed data. The chi-square or Fisher exact test was used to compare the proportions between the training and validation cohorts. Statistical analyzes were performed using the R software version 3.5.1 (R Foundation for Statistical Computing, Vienna, Austria) and SPSS version 25.0 (IBM Corporation, Armonk, NY).

## Results

### Clinical characteristics

Data of 1087 patients with COVID-19 who had been hospitalized in 47 regions of Sichuan and 2 regions of Wuhan, from January 2 to February 28, 2020, were obtained. Among these patients, 763 were assigned to the training cohort and 324 to the validation cohort. The demographic and clinical characteristics of the study cohort are presented in Table 1. A total of 97 patients were eventually admitted to the ICU (8.9%). The median age was 51 years (interquartile range, 37–65 years) in the training cohort and 50 years (interquartile range, 38–64 years) in the validation cohort. More than half of the patients were female subjects (training cohort, 51.4%; validation cohort, 52.5%). The most common symptoms were fever (training cohort, 63.0%; validation cohort, 66.0%), cough (training cohort, 59.8%; validation cohort, 65.1%), and fatigue (training cohort, 37.1%; validation cohort, 38.3%). The top 3 comorbidities were hypertension (training cohort, 24.8%; validation cohort, 23.1%), diabetes (training cohort, 12.7%; validation cohort, 10.2%), and cardiovascular system disease (training cohort, 7.5%; validation cohort, 6.2%).

Among the patients with ICU admission, most had a history of alcohol intake (training cohort, 79.4%; validation cohort, 86.2%), smoking (training cohort, 72.1%; validation cohort, 89.7%), and non-obesity (training cohort, 100%; validation cohort, 100%). Patients with ICU admission were older than those without ICU admission by a median of 6 years both in the training and validation cohorts. Most patients with ICU admission had systolic blood pressure > 100 mmHg, heart rate < 100 beats per minute, and temperature during admission between 36.2 °C and 38.0 °C. Nearly 90% of the patients among admission to ICU were exposed to Wuhan or other COVID-affected areas in the past 14 days.

### Selection of independent predictive factors

**Fig. 1:**
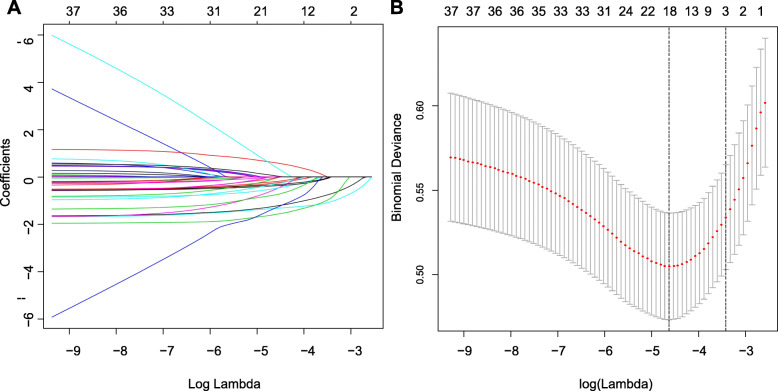
Selection of demographic and clinical features using the least absolute shrinkage and selection operator (LASSO) logistic regression model. **a**. Optimal parameter (lambda) selection in the LASSO model used fivefold cross-validation via minimum criteria. The partial likelihood deviance (binomial deviance) curve was plotted versus log (lambda). Dotted vertical lines were drawn at the optimal values by using the minimum criteria and the 1 standard error of the minimum criteria (the 1-SE criteria). **b**. Selection of optimal parameters (lambda) from the LASSO model using five-fold cross-validation and minimum criteria

On the basis of demographics, clinical signs on admission, clinical symptoms, clinical risk factors, and exposure to infection, 19 potential predictors with non-zero coefficients were selected in the LASSO logistic regression model (Fig. 1). The inclusion of these 19 variables in a logistic regression model resulted in 6 variables that were independently statistically significant predictors of admission to ICU.

The selected predictors were age, respiratory rate, systolic blood pressure, smoking status, fever, and CKD. The results of the logistic regression analysis are presented in Table 2.

**Table 2:** Logistic analysis of each factor’s ability in predicting the risk of ICU admission with COVID-19

	Prediction model		
	*β*	Odds ratio (95%CI)	*P*-value
Intercept	8.409	4485.633 (0.000-NA)	0.997
**Age < 65 years**	**− 1.650**	**0.192(0.102–0.356)**	**0.000**
Female	−0.548	0.578(0.312–1.055)	0.077
**Respiratory rate < 22**	**−1.516**	**0.220(0.120–0.403)**	**0.000**
**Systolic Blood Pressure > 100 mmHg**	**−1.466**	**0.231(0.067–0.966)**	**0.029**
**Non-Smoking**	**0.974**	**2.647(1.308–5.245)**	**0.006**
**Fever (No)**	**−0.912**	**0.402(0.186–0.808)**	**0.014**
Cough (No)	−0.172	0.842(0.438–1.583)	0.599
Dyspnea (No)	−0.489	0.613(0.325–1.177)	0.134
Fatigue (No)	−0.419	0.658(0.362–1.192)	0.166
Sore Throat (No)	−0.725	0.484(0.205–1.249)	0.112
Asthma (No)	14.989	32,340(0.000-NA)	0.984
Chronic Respiratory Disease (No)	−0.405	0.667(0.206–2.347)	0.509
**Chronic Kidney Disease (No)**	**−2.043**	**0.130(0.031–0.582)**	**0.005**
Cardiovascular System Disease (No)	−0.465	0.628(0.275–1.516)	0.283
Autoimmune Disease (No)	−1.132	0.322(0.075–1.544)	0.135
Hematological Disease (No)	−16.456	0.000(NA-Inf)	0.995
Stroke History (No)	−0.780	0.458(0.130–1.955)	0.251
Chronic Liver Disease (No)	0.041	1.042(0.361–3.854)	0.945
Without contact history of COVID-19	0.450	1.569(0.748–3.537)	0.252

### Building and validating a prediction nomogram model

**Fig. 2:**
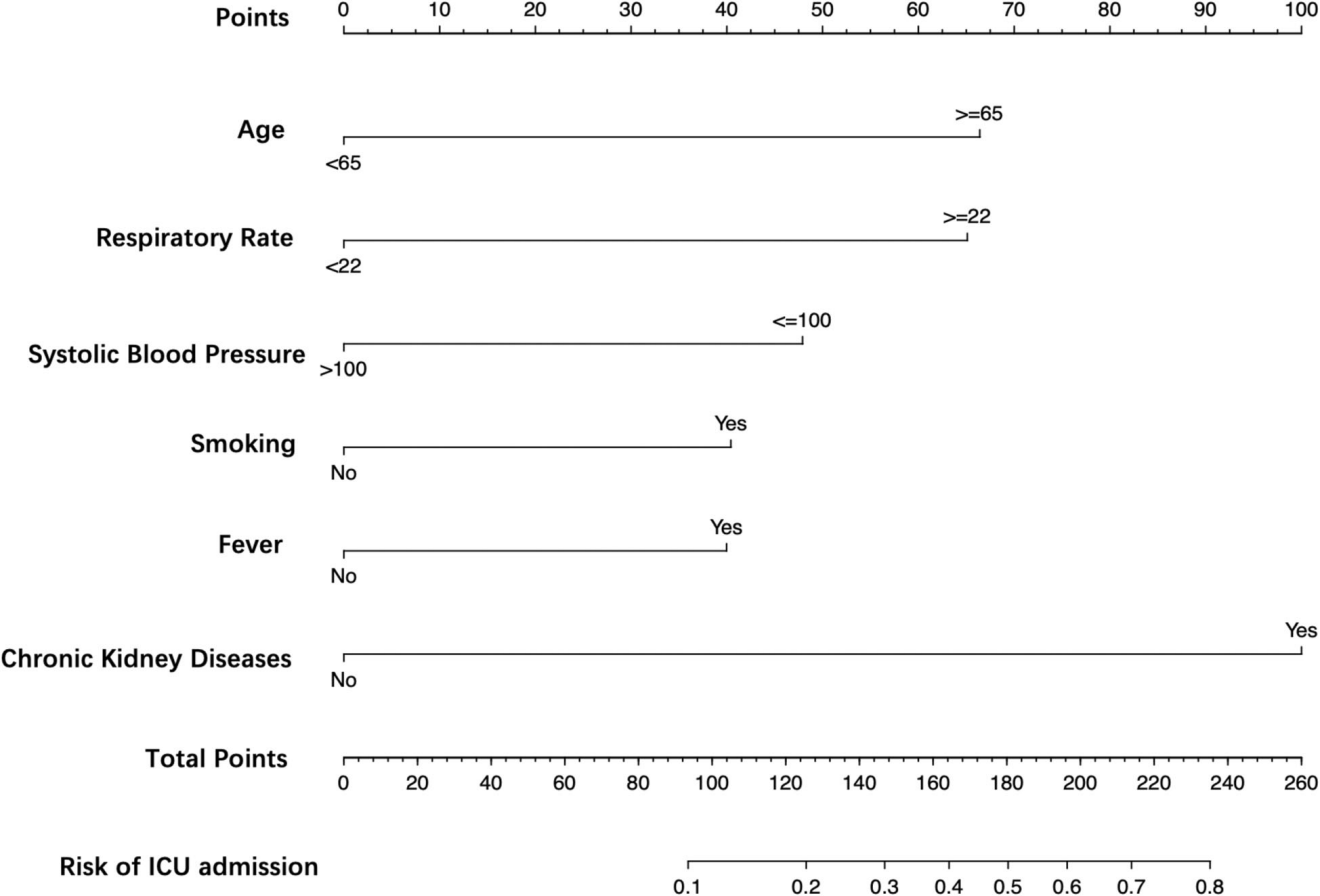
Development of a nomogram for predicting the risk of ICU admission in COVID-19 patients. The nomogram included age, respiratory rate, systolic blood pressure, smoking status, fever and chronic kidney disease. The nomogram summed the scores for each scale and variable. The total score on each scale indicated the risk of ICU admission

The nomogram used for predicting admission of patients with COVID-19 to ICU was formulated using the significant independent factors (age, respiratory rate, systolic blood pressure, smoking status, fever, and CKD). The nomogram revealed that the best predictors were CKD, age, and respiratory rate. Each variable was assigned a score according to the demographic and clinical features of an individual patient (Table 3), and the total score was computed by summing individual scores. The ICU admission probabilities were also obtained from the nomogram (Fig. 2).

**Table 3:** Score assignment for each variable included in the nomogram

Variables	Points
Age, years	
< 65	0
≥ 65	66
Respiratory Rate	
< 22	0
≥ 22	65
Systolic Blood Pressure, mmHg	
≤ 100	48
> 100	0
Smoking	
Former and/or Current	40
Never	0
Fever	
Yes	40
No	0
Chronic Kidney Disease	
Yes	0
No	100

**Fig. 3:**
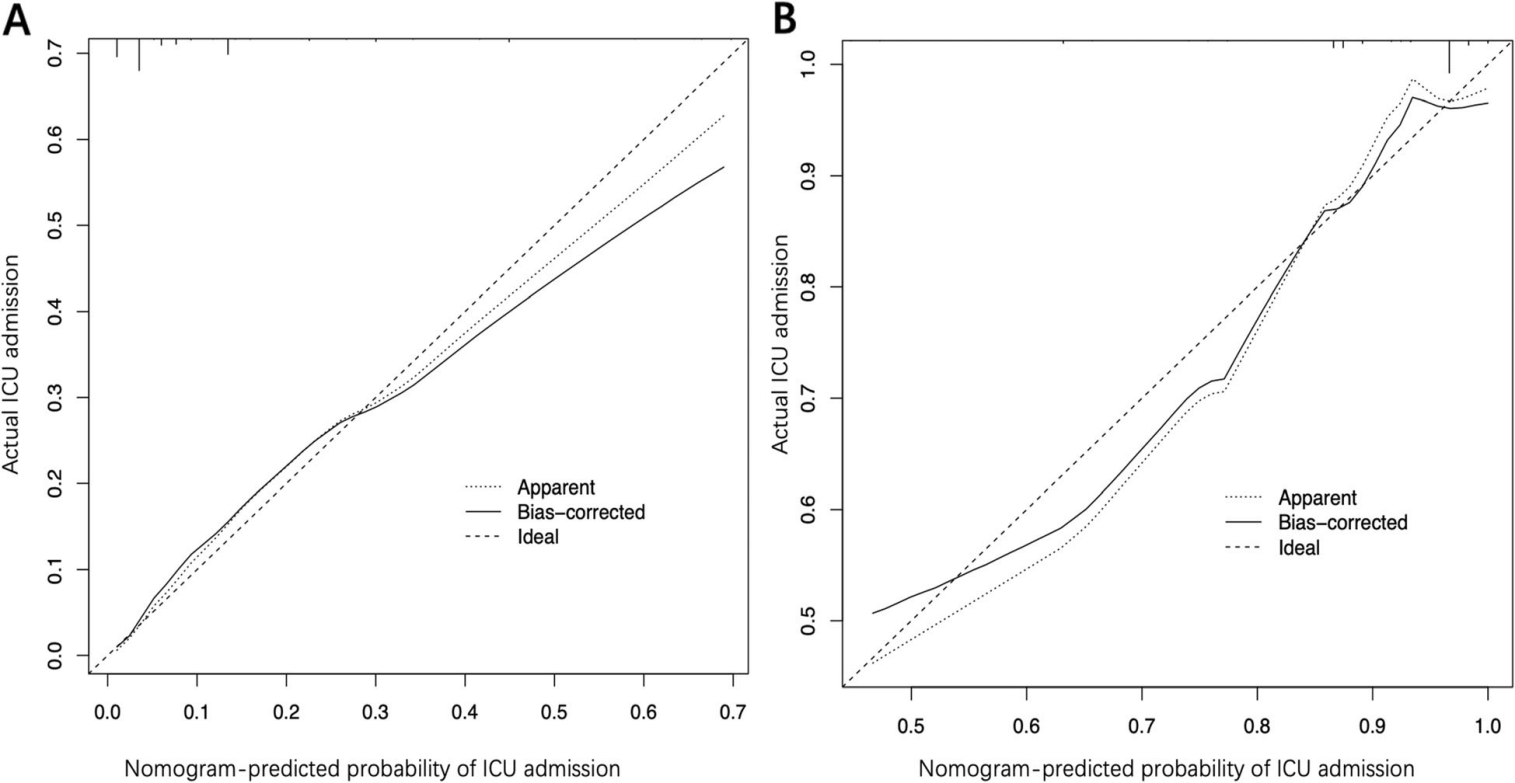
Calibration curves of the nomogram for predicting the risk of ICU admission in training (**a)** and validation cohort (**b**). Data on predicted and actual disease severity were plotted on the x- and y-axis, respectively. The diagonal dotted line indicates the ideal nomogram, in which actual and predicted probabilities are identical. The solid line indicates the actual nomogram, and a better fit to the dotted line indicates a better calibration

**Fig. 4:**
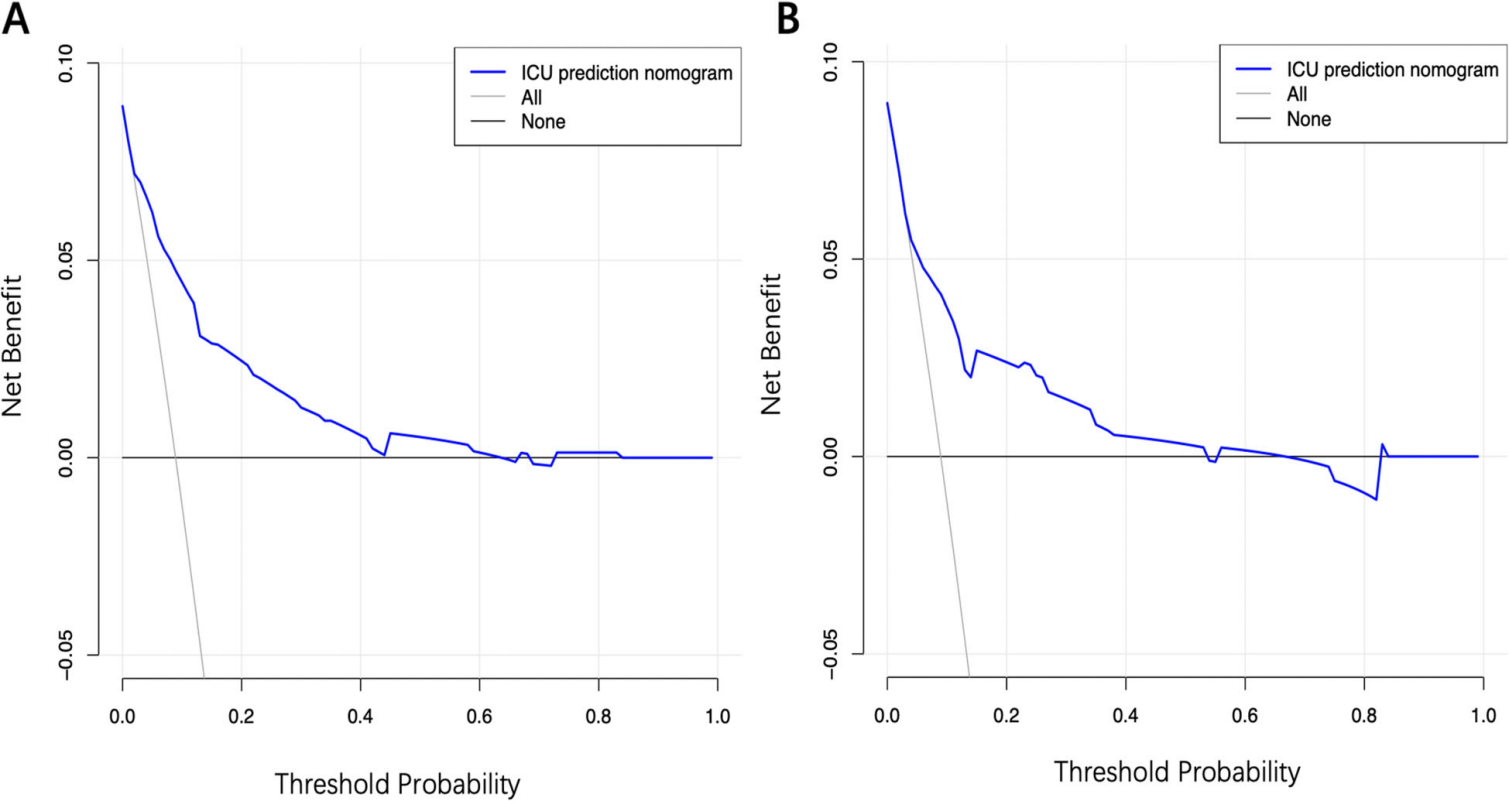
Decision curves of the nomogram predicting the risk of ICU admission in training (**a**) and validation cohort (**b**). The x-axis represents threshold probabilities and the y-axis measures the net benefit calculated by adding true positives and subtracting false positives

**Fig. 5:**
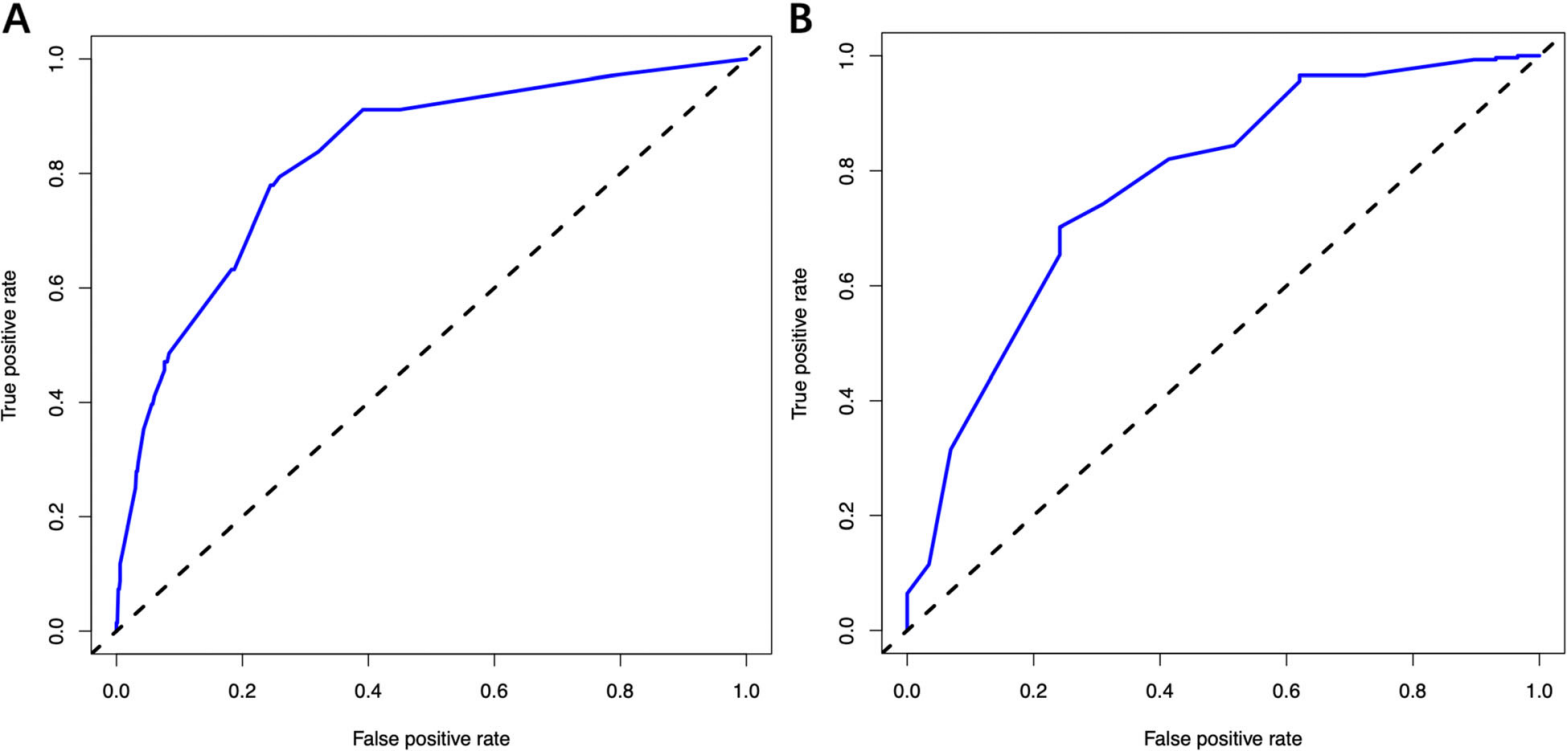
Receiver-operating characteristic curve of the nomogram for predicting the risk of ICU admission in training (**a**) and validation cohort (**b**)

The C-index of the nomogram was 0.829 (95% confidence interval [CI], 0.779–0.879) in the training cohort and 0.776 (0.684–0.868) in the validation cohort, implying the good discriminative ability of the model. The calibration plots of the nomogram revealed that the agreement between the predicted and observed disease severity was optimal in training and validation cohorts (Fig. 3). In addition, DCA revealed that the predictive model had significant net benefits for most threshold probabilities at different time points in training and validation cohorts, demonstrating the potential clinical benefit of the predictive model (Fig. 4). The AUC of the nomogram was 0.829 in the training cohort and 0.776 in the validation cohort, indicating the improved survival prediction compared with the nomogram model (Fig. 5).

## Discussion

Our study enrolled 1087 patients with COVID-19 who were registered from Sichuan and Hubei provinces’ health centers, where the outbreak risk levels were different. In the initial study, based on patient demographic and clinical characteristics obtained on the first admission, we established and validated a nomogram for predicting the risk for admission to ICU through LASSO, and logistic regression analyzes. The independently statistically significant factors included in the prediction model were age, respiratory rate, systolic blood pressure, smoking status, fever, and CKD. The validation of the model using different statistical methods demonstrated its optimal performance. As those factors can be obtained easily on admission, the nomogram is a convenient and valuable clinical warning tool to predict ICU admission of a patient with COVID-19, especially in the emergency department and even in a community health center.

Most patients with COVID-19 have mild disease with a good prognosis, but some patients may develop severe respiratory distress syndrome and have a poor prognosis [21]. To mitigate the burden on the healthcare system and provide the best care for patients, it is necessary to effectively predict the prognosis of the disease [22]. A predictive model that combines multiple variables or features to estimate the risk of poor outcomes of an infected person can assist the healthcare staff in classifying the patient’s disease severity when allocating limited medical resources [23]. Previous studies have reported prediction models for diagnosis and prognosis of COVID-19 and for detecting the risk of being admitted to a hospital for COVID-19. Chen et al. constructed a diagnosis prediction model with 10 clinical factors based on 136 participants [24]. Wang et al. enrolled 296 in-hospital patients with COVID-19 and developed a clinical model to predict the mortality of such patients [22]. Dong et al. developed a scoring model to predict the progression risk with COVID-19 pneumonia on the basis of 209 patients [25]. However, those proposed models are poorly reported and have a high risk of bias, raising concern of possible unreliable predictions when applied in daily practice for diagnosing. In a recent study, a risk score was reported to estimate the risk of critical illness of patients with COVID-19 based on 10 variables [26]. Although the study had a modest sample size and satisfying performance, the scoring system was complicated with some laboratory examination data that cannot be obtained before admission or quickly after admission. It is, therefore, necessary to develop and validate a convenient prediction model for healthcare staff or emergency staff that can be used quickly and easily. In our study, we constructed a warning model for predicting the risk of ICU admission on the basis of multi-center data from different cities and different severities of the outbreak in the Wuhan and Sichuan provinces. In our model, the independently statistically significant factors were age, respiratory rate, systolic blood pressure, smoking status, fever, and comorbidity with CKD, which could be obtained quickly, easily, practically, and reliably. This prediction model could be used in prehospital care or emergency department, allowing the medical staff to intervene at an early stage and determine their treatment location and the type of intervention. Statistically, our model demonstrated good discriminative ability and potential clinical benefit.

The model identified that comorbidities play a key role in the prognosis of patients with COVID-19. Cardiovascular system disease, especially hypertension, has been reported to be one of the most important independent risk factors [27]. In this study, we observed the patients with CKD were more likely to be admitted to the ICU, and that kidney disease was an independent risk factor for ICU admission of patients with COVID-19. This finding suggested that patients with a comorbidity of kidney disease on admission possibly had a high risk of deterioration [28, 29]. Previous studies revealed that kidney injury was associated with an increased risk of death in patients with influenza A virus subtype H1N1 and Severe acute respiratory syndrome (SARS). Multiple organ involvement, including the liver, gastrointestinal tract, and kidneys, has been reported during SARS in 2003 and very recently in patients with COVID-19 [30–33]. We hypothesized that such patients could have a proinflammatory state with functional defects in innate and adaptive immune-cell populations and were known to have a higher risk for upper respiratory tract infection and pneumonia. The 2019-nCoV itself may also cause kidney injury through multiple mechanisms: the 2019-nCoV may use angiotensin-converting enzyme 2 (ACE2) as a cell entry receptor and exert direct cytopathic effects on the kidney tissue. It has been reported ACE2 expression in the kidneys was nearly 100-fold higher than in the lungs [33]. Viral antigens or virus-induced specific immune effect mechanisms (specific T-cell lymphocytes or antibodies) and deposits of the immune complexes may damage the kidneys [34]. Early detection and treatment of renal abnormalities, including assessing the volume status and renal transplantation pressure, avoidance of nephrotoxic drugs, and adequate hemodynamic support, may help improve the vital prognosis of patients with COVID-19.

In most prognostic prediction models that have been published, older age, comorbidities, and increases in lactate dehydrogenase, lymphocyte, and C-reactive protein levels were the risk factors for poor prognosis [25]. Other indicators such as heart rate; breath rate; oxygen saturation; levels of procalcitonin, direct bilirubin, albumin, and D-dimer levels; activated partial thromboplastin time; glomerular filtration rate; and chest radiography abnormality have controversial conclusions [35, 36]. Our study also demonstrated that patients with COVID-19 infection who were older (especially > 65 years) had a worse prognosis than younger patients. In our study, fever (training cohort, 63.0%; validation cohort, 66.0%), cough (training cohort, 59.8%; validation cohort, 65.1%), and fatigue (training cohort, 37.1%; validation cohort, 38.3%) were the most common symptoms. However, among all the symptoms, only fever was an independent risk factor for prognosis, which is different from other studies. The difference in the inconsistencies of these models could be attributed to the risk of bias caused by the sample size and geographical differences of each model.

Our study has some limitations. First, the design was retrospective. Second, although the study is multi-center, the results cannot be generalized to other populations since the data is confined to just 2 places - Sichuan and Wuhan. Third, sample size limitation; future studies with larger sample sizes are warranted to validate our results. Fourth, some cases had incomplete data on symptoms, laboratory tests, and imaging examinations, given the variation in the structure of electronic databases across different participating hospitals and an urgent data extraction schedule. Fifth, severe patients were older than non-severe patients, and this difference in age may be a confounding factor. Sixth, we did not collect treatment-related data, which may be critical to the patient’s outcome. However, all patients received treatment in accordance with the guidelines issued by the National Health Commission of China.

## Conclusions

We established an early prediction model incorporating clinical characteristics that could be quickly obtained on hospital admission, even in community health centers. This model can be conveniently used to predict the individual risk for ICU admission of patients with COVID-19 and optimize the use of limited resources.

## Data Availability

The datasets used and/or analyzed in the present study are available from the corresponding author on reasonable request.

## Abbreviations

COVID-19: Coronavirus disease 2019
ICU: Intensive care unit
PSI: Pneumonia severity index
REMS: Rapid emergency medicine score
MEWS: Modified early warning score
NPV: Negative predictive value
LASSO: Least absolute shrinkage, and selection operator
AUC: Area under the curve
RT-PCR: Real-time reverse-transcriptase polymerase chain reaction
OR: Odds ratio
C-index: Concordance index
DCA: Decision curve analysis
ROC: Receiver-operating-characteristic
CI: Confidence interval
SARS: Severe acute respiratory syndrome
MERS: The Middle East respiratory syndrome

## References

[1] Hui DS, Esam IA, Madani TA, Ntoumi F, Kock R, Dar O, Ippolito G, TD MH, Memish ZA, Drosten C (2020). The continuing 2019-nCoV epidemic threat of novel coronaviruses to global health - the latest 2019 novel coronavirus outbreak in Wuhan, China. Int J Infect Dis.

[2] Zhou P, Yang XL, Wang XG, Hu B, Zhang L, Zhang W, Si HR, Zhu Y, Li B, Huang CL (2020). A pneumonia outbreak associated with a new coronavirus of probable bat origin. Nature.

[3] World Health Organization. Coronavirus Disease 2019 (COVID-19) Situation Report-123. 23 May 2020 (2020). In*.*.

[4] Lauer SA, Grantz KH, Bi Q, Jones FK, Zheng Q, Meredith HR, Azman AS, Reich NG, Lessler J (2020). The incubation period of coronavirus disease 2019 (COVID-19) from publicly reported confirmed cases: estimation and application. Ann Intern Med.

[5] Wu Z, McGoogan JM. Characteristics of and important lessons from the coronavirus disease 2019 (COVID-19) outbreak in China: summary of a report of 72314 cases from the Chinese Center for Disease Control and Prevention. JAMA. 2020;323(13):1239–42. 10.1001/jama.2020.2648 32091533

[6] Dietz W, Santos-Burgoa C (2020). Obesity and its Implications for COVID-19 Mortality. Obesity (Silver Spring).

[7] Wynants L, Van Calster B, Bonten MMJ, Collins GS, Debray TPA, De Vos M, Haller MC, Heinze G, Moons KGM, Riley RD (2020). Prediction models for diagnosis and prognosis of covid-19 infection: systematic review and critical appraisal. BMJ.

[8] Satici C, Demirkol MA, Sargin Altunok E, Gursoy B, Alkan M, Kamat S, Demirok B, Surmeli CD, Calik M, Cavus Z (2020). Performance of pneumonia severity index and CURB-65 in predicting 30-day mortality in patients with COVID-19. Int J Infect Dis.

[9] Hu H, Yao N, Qiu Y (2020). Comparing rapid scoring Systems in Mortality Prediction of critically ill patients with novel coronavirus disease. Acad Emerg Med.

[10] Fu L, Wang B, Yuan T, Chen X, Ao Y, Fitzpatrick T, Li P, Zhou Y, Lin YF, Duan Q (2020). Clinical characteristics of coronavirus disease 2019 (COVID-19) in China: a systematic review and meta-analysis. J Inf Secur.

[11] Organization WH: Coronavirus disease (COVID-19) technical guidance: laboratory testing for 2019-nCoV in humans. In*.*; 2019.

[12] Organization. WH: Clinical management of severe acute respiratory infection when novel coronavirus (2019-nCoV) infection is suspected: interim guidance**.** In*.*, January 28 edn; 2020.

[13] Lei Z, Li J, Wu D, Xia Y, Wang Q, Si A, Wang K, Wan X, Lau WY, Wu M (2016). Nomogram for preoperative estimation of microvascular invasion risk in hepatitis B virus-related hepatocellular carcinoma within the Milan criteria. JAMA Surg.

[14] Attiyeh MA, Fernandez-Del Castillo C, Al Efishat M, Eaton AA, Gonen M, Batts R, Pergolini I, Rezaee N, Lillemoe KD, Ferrone CR (2018). Development and validation of a multi-institutional preoperative Nomogram for predicting grade of dysplasia in Intraductal papillary mucinous neoplasms (IPMNs) of the pancreas: a report from the pancreatic surgery consortium. Ann Surg.

[15] Metlay JP, Waterer GW, Long AC, Anzueto A, Brozek J, Crothers K, Cooley LA, Dean NC, Fine MJ, Flanders SA (2019). Diagnosis and treatment of adults with community-acquired pneumonia an official clinical practice guideline of the American Thoracic Society and Infectious Diseases Society of America. Am J Resp Crit Care.

[16] Gao HN, Lu HZ, Cao B, Du B, Shang H, Gan JH, Lu SH, Yang YD, Fang Q, Shen YZ (2013). Clinical findings in 111 cases of influenza a (H7N9) virus infection. N Engl J Med.

[17] Sauerbrei W, Royston P, Binder H (2007). Selection of important variables and determination of functional form for continuous predictors in multivariable model building. Stat Med.

[18] Friedman J, Hastie T, Tibshirani R (2010). Regularization paths for generalized linear models via coordinate descent. J Stat Softw.

[19] Kidd AC, McGettrick M, Tsim S, Halligan DL, Bylesjo M, Blyth KG. Survival prediction in mesothelioma using a scalable Lasso regression model: instructions for use and initial performance using clinical predictors. BMJ Open Respir Res. 2018;5(1):e000240. 10.1136/bmjresp-2017-000240 PMC5812388 29468073

[20] Alba AC, Agoritsas T, Walsh M, Hanna S, Iorio A, Devereaux PJ, McGinn T, Guyatt G (2017). Discrimination and calibration of clinical prediction models Users' guides to the medical literature. Jama-J Am Med Assoc.

[21] Huang C, Wang Y, Li X, Ren L, Zhao J, Hu Y, Zhang L, Fan G, Xu J, Gu X (2020). Clinical features of patients infected with 2019 novel coronavirus in Wuhan, China. Lancet.

[22] Wang K, Zuo P, Liu Y, Zhang M, Zhao X, Xie S, Zhang H, Chen X, Liu C. Clinical and laboratory predictors of in-hospital mortality in patients with COVID-19: a cohort study in Wuhan, China. Clin Infect Dis. 2020:ciaa538. 10.1093/cid/ciaa538 PMC7197616 32361723

[23] Hong Y, Wu X, Qu J, Gao Y, Chen H, Zhang Z (2020). Clinical characteristics of coronavirus disease 2019 and development of a prediction model for prolonged hospital length of stay. Ann Transl Med.

[24] Chen X, Tang Y, Mo Y, Li S, Lin D, Yang Z, Yang Z, Sun H, Qiu J, Liao Y, et al. A diagnostic model for coronavirus disease 2019 (COVID-19) based on radiological semantic and clinical features: a multi-center study. Eur Radiol. 2020. 10.1007/s00330-020-06829-2 PMC7160614 32300971

[25] Ji D, Zhang D, Xu J, Chen Z, Yang T, Zhao P, Chen G, Cheng G, Wang Y, Bi J, et al. Prediction for progression risk in patients with COVID-19 pneumonia: the CALL score. Clin Infect Dis. 2020. 10.1093/cid/ciaa414 PMC7184473 32271369

[26] Liang W, Liang H, Ou L, Chen B, Chen A, Li C, Li Y, Guan W, Sang L, Lu J, et al. China Medical Treatment Expert Group for COVID-19. Development and validation of a clinical risk score to predict the occurrence of critical illness in hospitalized patients with COVID-19. JAMA Intern Med. 2020;180(8):1081–9. 10.1001/jamainternmed.2020.2033 PMC7218676 32396163

[27] Guzik TJ, Mohiddin SA, Dimarco A, Patel V, Savvatis K, Marelli-Berg FM, Madhur MS, Tomaszewski M, Maffia P, D'Acquisto F, et al. COVID-19 and the cardiovascular system: implications for risk assessment, diagnosis, and treatment options. Cardiovasc Res. 2020;116(10):1666–87. 10.1093/cvr/cvaa106 PMC7197627 32352535

[28] Cheng YC, Luo R, Wang K, Zhang M, Wang ZX, Dong L, Li JH, Yao Y, Ge SW, Xu G (2020). Kidney disease is associated with in -hospital death of patients with COVID-19. Kidney Int.

[29] Trujillo H, Caravaca-Fontán F, Sevillano Á, Gutiérrez E, Caro J, Gutiérrez E, Yuste C, Andrés A, Praga M. SARS-CoV-2 infection in hospitalized patients with kidney disease. Kidney Int Rep. 2020;5(6):905–9. 10.1016/j.ekir.2020.04.024 PMC7194060 32363253

[30] Chu KH, Tsang WK, Tang CS, Lam MF, Lai FM, To KF, Fung KS, Tang HL, Yan WW, Chan HW (2005). Acute renal impairment in coronavirus-associated severe acute respiratory syndrome. Kidney Int.

[31] Kumar A, Zarychanski R, Pinto R, Cook DJ, Marshall J, Lacroix J, Stelfox T, Bagshaw S, Choong K, Lamontagne F (2009). Critically ill patients with 2009 influenza a(H1N1) infection in Canada. JAMA.

[32] Jung JY, Park BH, Hong SB, Koh Y, Suh GY, Jeon K, Koh SO, Kim JY, Cho JH, Choi HS (2011). Acute kidney injury in critically ill patients with pandemic influenza a pneumonia 2009 in Korea: a multi-center study. J Crit Care.

[33] Guan WJ, Liang WH, Zhao Y, Liang HR, Chen ZS, Li YM, Liu XQ, Chen RC, Tang CL, Wang T, et al. China Medical Treatment Expert Group for COVID-19. Comorbidity and its impact on 1590 patients with COVID-19 in China: a nationwide analysis. Eur Respir J. 2020;55(5):2000547. 10.1183/13993003.00547-2020 PMC7098485 32217650

[34] Naicker S, Yang CW, Hwang SJ, Liu BC, Chen JH, Jha V (2020). The novel coronavirus 2019 epidemic and kidneys. Kidney Int.

[35] Bai Z, Gong Y, Tian X, Cao Y, Liu W, Li J. The rapid assessment and early warning models for COVID-19. Virol Sin. 2020;35(3):272–9. 10.1007/s12250-020-00219-0 PMC7110270 32239446

[36] Huang J, Cheng A, Kumar R, Fang Y, Chen G, Zhu Y, Lin S. Hypoalbuminemia predicts the outcome of COVID-19 independent of age and co-morbidity. J Med Virol. 2020:10.1002/jmv.26003. 10.1002/jmv.26003 PMC7273060 32406952

